# New Lower-Limb Gait Asymmetry Indices Based on a Depth Camera

**DOI:** 10.3390/s150304605

**Published:** 2015-02-24

**Authors:** Edouard Auvinet, Franck Multon, Jean Meunier

**Affiliations:** 1 M2S, University Rennes 2, ENS Rennes, Campus de Ker lann, Avenue Robert Schuman, Bruz 35170, France; E-Mail: franck.multon@irisa.fr; 2 Département d’informatique et de recherche opérationnelle, Université de Montréal, C.P. 6128, succ. Centre-ville, Montréal H3C 3J7, QC, Canada; E-Mail: meunier@iro.umontreal.ca; 3 Inria, Campus Universitaire de Beaulieu, Rennes 35052, France

**Keywords:** gait, asymmetry, Kinect, depth camera

## Abstract

Background: Various asymmetry indices have been proposed to compare the spatiotemporal, kinematic and kinetic parameters of lower limbs during the gait cycle. However, these indices rely on gait measurement systems that are costly and generally require manual examination, calibration procedures and the precise placement of sensors/markers on the body of the patient. Methods: To overcome these issues, this paper proposes a new asymmetry index, which uses an inexpensive, easy-to-use and markerless depth camera (Microsoft Kinect™) output. This asymmetry index directly uses depth images provided by the Kinect™ without requiring joint localization. It is based on the longitudinal spatial difference between lower-limb movements during the gait cycle. To evaluate the relevance of this index, fifteen healthy subjects were tested on a treadmill walking normally and then via an artificially-induced gait asymmetry with a thick sole placed under one shoe. The gait movement was simultaneously recorded using a Kinect™ placed in front of the subject and a motion capture system. Results: The proposed longitudinal index distinguished asymmetrical gait (*p* < 0.001), while other symmetry indices based on spatiotemporal gait parameters failed using such Kinect™ skeleton measurements. Moreover, the correlation coefficient between this index measured by Kinect™ and the ground truth of this index measured by motion capture is 0.968. Conclusion: This gait asymmetry index measured with a Kinect™ is low cost, easy to use and is a promising development for clinical gait analysis.

## 1. Introduction

In pathological gait, significant differences in kinematic and kinetic parameters can be observed between the left and right human lower extremities [1]. Gait asymmetry can consequently be used to identify pathology and track recovery. To do so, various asymmetry indices and ratios have been developed [2]. These measurements could provide insight into the efficiency of the treatment of people with asymmetric gait, whatever the origin, e.g., cerebral palsy [3], stroke [4], hip arthritis [5] and leg length discrepancy [6].

As reported in [4], most of these indices rely on a simple comparison between left and right variables, such as step length and step duration, joint angles or ground reaction force. However, they provide only a unique value for the whole gait cycle without considering the possible evolution of gait asymmetry that may occur within the cycle. Moreover, measuring the variation of asymmetry inside the gait cycle would allow one to identify when the maximum of asymmetry occurred during the gait cycle.

Moreover, most of the current indices rely on data provided by sensors or markers that are placed on the body, such as optical motion capture [3] or inertial systems [7,8]. These methods require expertise for marker/sensor placement, calibration and manual editing of the data, which involves recruiting trained staff and requires time for measurement preparation and analysis. Gaitrite [4] or a force plate can also be used to measure spatiotemporal parameters in an almost automatic manner. However, they fail to give joint coordination information at each time of the gait cycle and could underestimate asymmetry due to compensation strategies along the kinematic chain [9]. Carroza *et al*. [10] proposed a method to use 16 video cameras to reconstruct the volume of the subject in order to compute the joint orientation. However, this method still requires a calibration stage. To sum-up, an index that could estimate gait asymmetry evolution during the gait cycle, without markers or sensors on the patient’s body, without calibration and without manual editing does not exist yet.

Depth cameras have recently been introduced in the public market through the increase of the Microsoft Kinect™ sensor usage. It has rapidly been used to measure 3D human motion, as it provides a low-cost, markerless and calibrationless system [11]. Paolini proposed a method in [12] that uses the Kinect™ to measure foot position on a treadmill, but a marker on each foot is still required. Two methods have been introduced to measure spatiotemporal gait parameters without markers (mainly step length and duration) using a Kinect™. Stone and Skubic [13] computed the subject’s centroid in depth images to deduce the step length and the lower part of the legs to detect gait cycle. Clark *et al.* [11] computed gait parameters thanks to a 3D skeleton fitted to the depth maps [14]. However, for both methods, inaccuracies can appear because of possible confusion between the feet and the ground during the contact phase due to the Kinect™ noisy output. This leads to inaccurate computation of the spatiotemporal gait parameters, which is key information in any asymmetry assessment. Moreover, none of them addressed gait asymmetry measurements, especially during the step. Furthermore, this use of the skeleton for gait analysis on a treadmill has been evaluated in [15]. The hip angle and knee angle measurements were compared with reference motion capture system measurements. The hip angle correlation was lower than 0.3, and the knee angle was mainly less than 0.8, which could lead to erroneous gait parameter estimation. According to [15], the skeleton information would need some improvement in sensitivity before being clinically usable. Moreover, the skeleton-fitting algorithm was trained with healthy subjects and could possibly fail to deliver accurate information in the case of impaired subjects.

**Figure 1:**
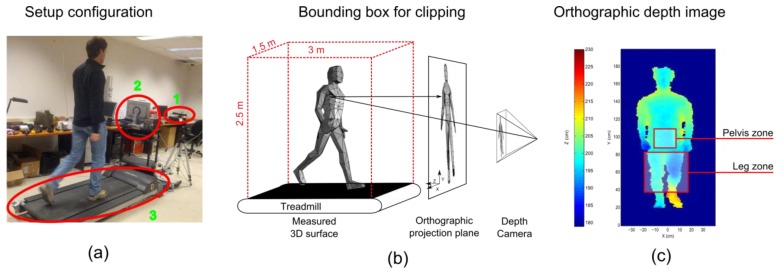
(**a**) Representation of the experimental set-up featuring the depth camera (1), the recording computer (2) and the treadmill (3); (**b**) representation of the orthographic projection operation and the bounding box in red, chosen to isolate the points belonging to the subject; (**c**) the orthographic depth image showing the parts of the body defining the limits of the regions of interest used for the registration operation and asymmetry indices’ computation.

To overcome these limitations, this paper proposes a new index to assess gait asymmetry inside the step, using depth images with no skeleton fitting. This asymmetry index is based on spatial differences between the lower limb positions at each instant of the step. It uses information recorded by a depth camera Kinect™ placed in front of the subject walking on a treadmill, as shown Figure 1a.

## 2. Method

The key concept of the proposed asymmetry index is to compare the spatial position of the left and the right legs at comparable times within their respective step cycle (*i.e.*, at the same percentage of time in each step cycle). 

**Figure 2:**
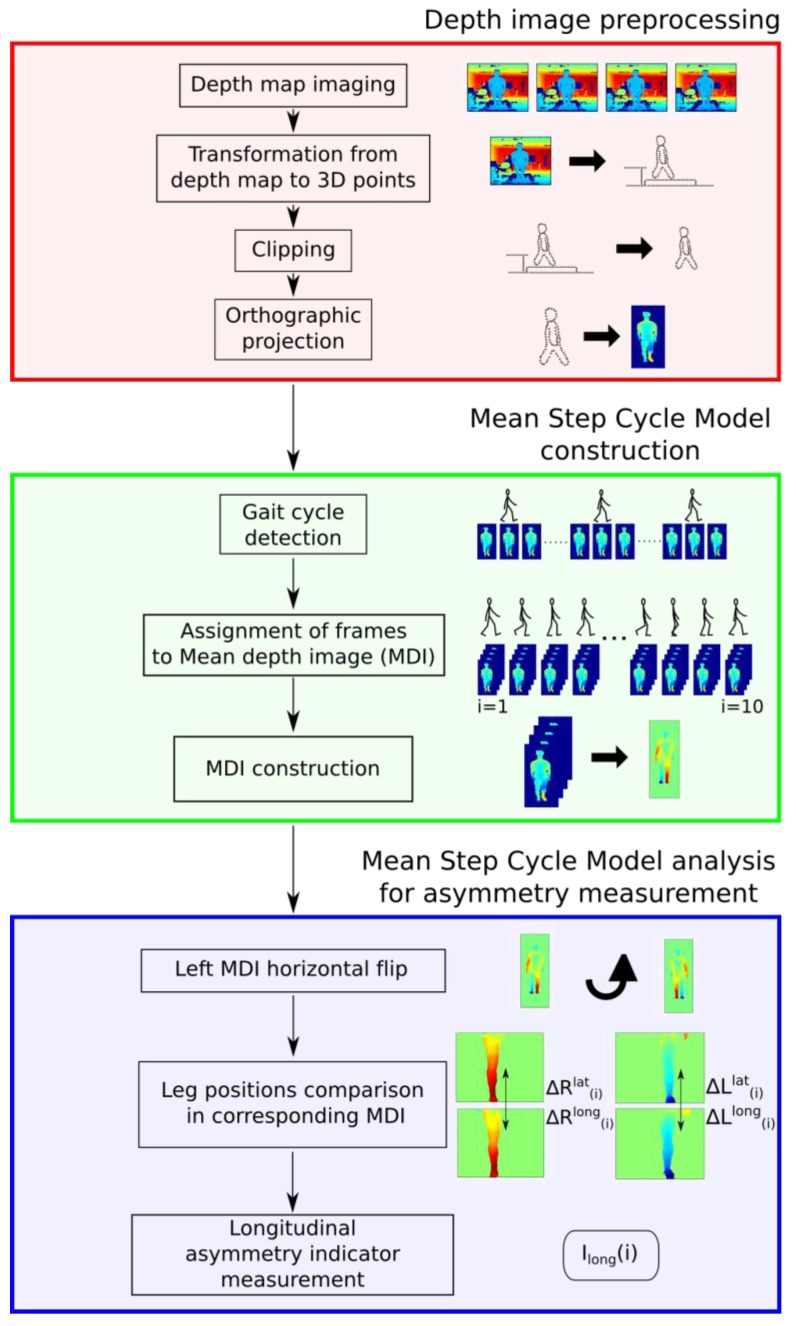
Representation of the method for measuring asymmetry indices of lower limbs during the step cycle. In the red box are the depth image pretreatment operations. In the green box are the mean step cycle model construction steps. Finally, in the blue box are the different operations to construct the asymmetry indices.

Kinect™ sensors are based on depth images that could be processed by the Shotton algorithm [14] to segment body parts and estimate joint centers. We assume that this post-processing may lead to less accurate results in gait asymmetry measurements compared to the use of raw depth images. A mean step cycle model is introduced in this paper in order to obtain a more representative and reliable sequence of depth images before asymmetry analysis. The overall process is depicted in Figure 2. The details are given in the following sections.

### 2.1. Raw Depth Image Preprocessing

Four stages are needed to process the raw depth images provided by the depth camera before computing the asymmetry index, as shown in Figure 2. Let us consider these stages in detail.

#### 2.1.1. Transformation from Raw Depth Images to 3D Points 

The depth camera is considered as a pinhole camera with its optical axis intersecting the center of the image plane and the focal length $f=575.82\;px\cdot m^{-1}$ given by the manufacturer. Then, horizontal (*X*), vertical (*Y*) and depth (*Z*) coordinates of 3D points are reconstructed according to the following equations using the depth information z returned for each pixel (x, y), where w and h are the width and height of the depth image (in pixels), respectively.

(1) $$\begin{array}{ccc} X=(x-\frac{w}{2})\frac{z}{f} & Y=(y-\frac{w}{2})\frac{z}{f} & Z=z \end{array}$$

#### 2.1.2. Clipping 

Because the subject is above the treadmill, only points located in the 2.5 m × 3 m × 1.5 m (height × depth × width) bounding box above the treadmill belt surface (depicted in Figure 1b) are associated with the subject and kept for further processing.

#### 2.1.3. Orthographic Projection 

The depth information returned by the Kinect™ is provided in a projective representation. As shown in Equation (1), the lateral and height position of a point in this projective representation depend on its depth. Hence, two points in space with identical heights, but different depths will have different vertical positions in the projective image. Without projection correction, the same ankle joint could be displayed at two different heights, depending on its depth, with a difference of up to 60 pixels. To ensure that left and right body parts are correctly compared in the following method, 3D surfaces are orthographically projected on a plane perpendicular to the optical axis of the depth camera, as illustrated in Figure 1b. The spatial resolution of this orthogonal image was set to 0.5 cm·px^−1^ which is close to the resolution of the depth camera (Kinect™) when the subject is 2 m away from the sensor. The depth value of each pixel was computed using the inverse distance interpolation, as described by Shepard [16]. As an output, this preprocessing stage transforms each raw projective view in orthographic projections of the 3D surface of the subject (depth images (DI)).

#### 2.1.4. Lower-Limb Region of Interest and Pelvis Zone

The asymmetry index presented in this paper focuses on lower-limb movements. This lower-limb region is called the leg zone. This leg zone is defined as the volume between the lower leg zone parameter at 0.22 H and the upper leg zone parameter at 0.47 H, where H is the height of the subject. These parameter value estimations were done posteriorly with our dataset in order to maximize the correlation between Kinect™ measurements and ground truth obtained with reference to motion capture systems. In the same way, to ensure correct comparison of left and right legs at various times in the gait cycles, we need to displace the pelvis location (origin of the kinematic chain) to a unique position, *i.e.*, the same distance to the camera. To this end, the pelvis zone is defined by a 20-cm square centered on the pelvis, the position of which is estimated at 0.61 H, according to anthropometrical tables [17]. 

### 2.2. Mean Step Cycle Model Computation

The Kinect™ measurements have a random error [18] with a standard deviation up to 1 cm when the subject is 2.5 m from the sensor. This error could result in a decreased accuracy of the asymmetry estimation. The mean step cycle model (MSCM) aims at helping to decrease the impact of the sensor’s noise on the computation of the index. It consists of computing a sequence of mean depth images (MDI) representing the most representative (averaged) gait cycle for each subject. This is performed in three main stages.

#### 2.2.1. Step Cycle Detection 

Firstly, it is necessary to determine the beginning and end moment of each step cycle in order to extract a set of step cycles. Traditionally, step cycles are defined as two successive heel strikes of the right and left feet. Unfortunately, heel positions are difficult to track in a depth image, because the points of the feet could be confused with the treadmill belt. We therefore used the cyclic longitudinal distance between the knees to estimate heel strike and identify steps, as described in [19].

#### 2.2.2. Assignation of Frame to Mean Depth Image

Once these events are identified, each step is divided into ten 10%-long intervals. The MSCM is also cut into 10 intervals. The *i*-*th* MDI of the MSCM is the average image of the DI belonging to the *i*-*th* interval of all of the recorded steps. Now, consider the details of this averaging process.

#### 2.2.3. Mean Depth Image Construction 

The objective here is to compute a representative mean depth image MDI_(s,i)_ for each step side *s* (s∈{R,L} for left and right) and for each interval *I* (*i* = 1,...,10) of the step, which corresponds to the average of the depth images DI_(s,c,i)_ for all of the recorded steps *c* (*c* = 1,...,n). This averaging process requires a registering procedure in order to suppress the lateral and longitudinal movements of the subject on the treadmill that are not relevant. To this end, we consider each first step DI_(s,1,i)_ for each interval *i* and step side *s* as a reference depth image. 

Consequently, each DI_(s,c,i)_ can be laterally registered by computing the lateral shift ${∆}_{x(s,c,i)}$ that maximizes the cross-correlation between DI_(s,c,i)_ and the corresponding reference DI_(s,1,i)_ leg of each step *c*. This process is only applied on the pixels belonging to the leg zones. ${∆}_{x(s,c,i)}$ is computed with Equation (2), where *x* and *y* are, respectively, the lateral and vertical coordinates in the depth image, and *y* lies between 0.22 H and 0.47 H.

(2) $${∆}_{x_{\left(s,c,i\right)}}=\underset{\partial x}{argmax}\underset{x}{\sum }\overset{y=0.47H}{\underset{y=0.22H}{\sum }}DI_{\left(s,c,i\right)}\left(x+\partial x,y\right)*DI_{\left(s,1,i\right)}\left(x,y\right)$$

The longitudinal registration is done by subtracting the mean depth of the pelvic zone from all of the DI_(s,c,i)_.

MDIs for each step side *s* and each interval *i* are computed using the Equation (3).

(3) $$MDI_{\left(s,i\right)}\left(x,y\right)=\frac{\sum_{c=1,\mathrm{...},n}DI_{\left(s,c,i\right)}\left(x+{∆}_{x_{\left(s,c,i\right)}},y\right)-{∆}_{z_{\left(s,c,i\right)}}}{n}$$

where ${∆}_{z_{\left(s,c,i\right)}}$ is the mean depth of the pelvis for step side *s*, step *c* and interval *i*. The final MSCM is consequently composed of 10 MDI for the right step and 10 MDI for the left step, denoted respectively MDI_(R,i)_ and MDI_(L,i)_, *i* = 1,…,10. Finally, in order to allow the horizontal flip, the subject is laterally centered. This is done in each MDI with the computation of the center of the subject and the application of a lateral translation in order to align the center of the subject with the center of the image.

### 2.3. Asymmetry Index Based on the Mean Gait Cycle Model 

The goal of this phase is to compute the index, which quantifies the longitudinal difference between the left and the right leg movements within the step. To this end, we use the MSCM as the representative of the step of the subject. The analysis of the MSCM begins with the selection of corresponding intervals *i* (*i* = 1,…,10) in the right and left steps. If the gait is symmetric, we expect leg positions of the right step in MDI_(R,i)_ to be similar to those of the left step in MDI_(L,i)_ after being horizontally flipped (denoted fMDI_(L,i)_), for each interval *i*. In the following, the right step is used as a reference, and the left step is compared to this reference. In each MDI_(R,i)_ and fMDI_(L,i)_, the right and left legs are identified with a two-class K-means algorithm, as described in Auvinet *et al.* [19]. We will denote leftleg(.) (resp. rightleg(.)) as the function that returns the points belonging to the left leg (resp. right leg) in the depth images, which is provided as an input.

**Figure 3:**
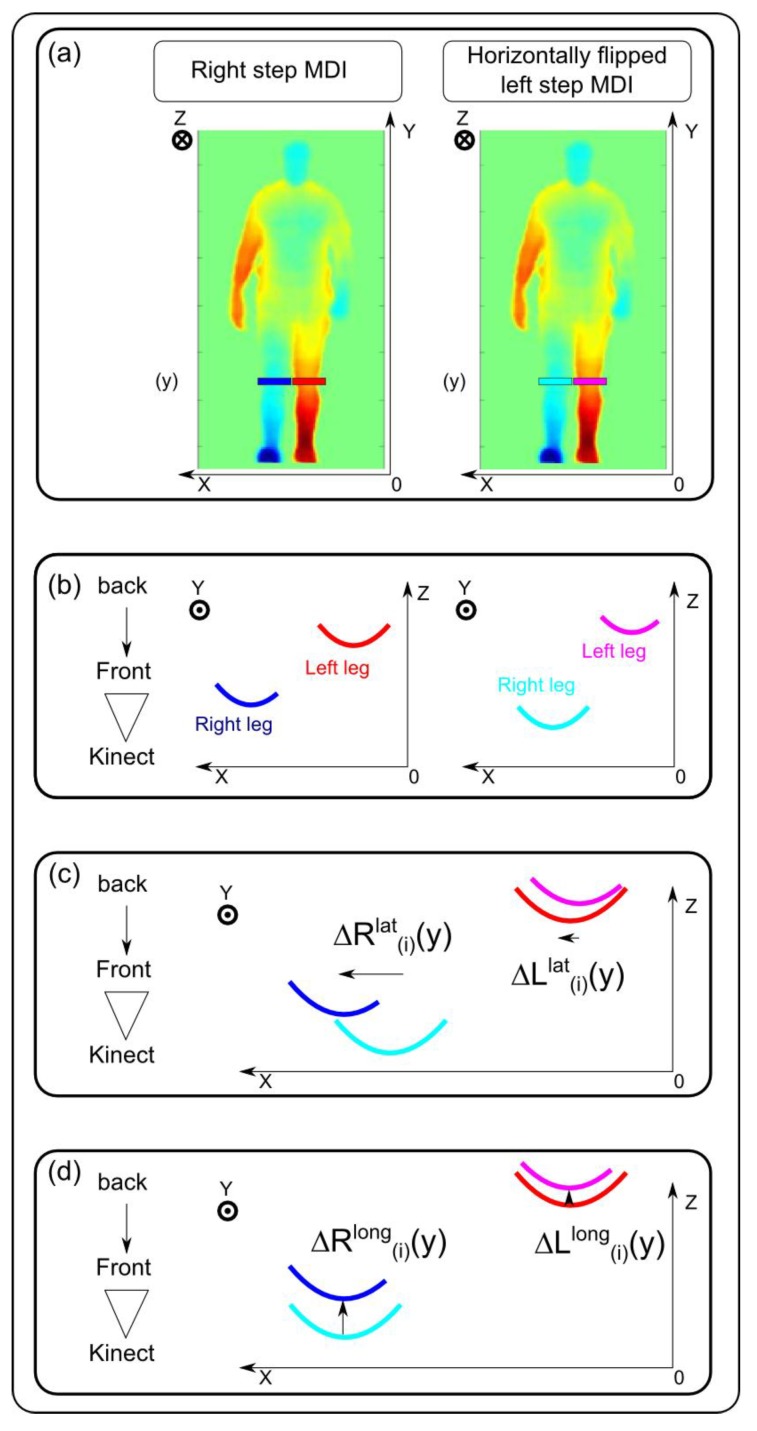
(**a**) Position of the horizontal lines (y) selected in the legs from right step MDI(*i*) and corresponding left step fMDI(*i*), for lateral and longitudinal asymmetry evaluation; (**b**) representation of these horizontal lines in the transverse plane. Each leg depth curve is plotted in red and blue for, respectively, the right and left legs of the right step MDI(*i*) and cyan and magenta for, respectively, the right and left legs of the left step fMDI(*i*). (**c**) Lateral differences $∆R_{\left(i\right)}^{lat}(y)$ and $∆L_{\left(i\right)}^{lat}(y)$ are calculated with a lateral inter-correlation of corresponding leg depth curves; (**d**) once the legs of the left step are laterally registered on the legs of the right step, the depth differences $∆R_{\left(i\right)}^{long}(y)$ and $∆L_{\left(i\right)}^{long}(y)$ are computed by averaging the depth difference between depth curves.

Let us recall that the resulting index aims at assessing gait asymmetry by computing the spatial difference between the two legs at a comparable time, at each interval *i* of the step. It consists of computing the positional difference between the corresponding legs in MDI_(R,i)_ and the corresponding fMDI_(L,i)_, as shown in Figure 3. These differences are computed for each height *y* of the leg zone (*i.e.*, *y* between 0.22 H and 0.47 H) and at each interval *i* of the step (*i.e.*, *i* = 1,...,10). We propose to compute this spatial difference along the longitudinal axis to provide the user with information concerning longitudinal asymmetry. This therefore leads to the I_long_ (longitudinal asymmetry index). Let us consider now the computational details for this index.

Before computing the longitudinal difference between corresponding legs, it is necessary to displace each leg of fMDI_(L,i)_ laterally in order to align them with their corresponding leg of MDI_(R,i)_. This lateral difference between the right leg (resp. left leg) of the reference step from MDI_(R,i)_ and the corresponding leg of the compared step from fMDI_(L,i)_ is denoted $∆R_{\left(i\right)}^{lat}$ (resp. $∆L_{\left(i\right)}^{lat}$). These lateral differences are computed, at each height *y* of the leg zone and interval *i* in the step, in order to maximize the correlation between corresponding legs:

(4) $$∆R_{\left(i\right)}^{lat}(y)=\underset{\partial x}{argmax}\underset{x}{\sum }rightleg\left(MDI_{\left(R,i\right)}\left(x,y\right)\right)*rightleg\left(fMDI_{\left(L,i\right)}\left(x+\partial x,y\right)\right)$$

(5) $$∆L_{\left(i\right)}^{lat}(y)=\underset{\partial x}{argmax}\underset{x}{\sum }leftleg\left(MDI_{\left(R,i\right)}\left(x,y\right)\right)*leftleg\left(fMDI_{\left(L,i\right)}\left(x+\partial x,y\right)\right)$$

Once corresponding legs are laterally aligned, the longitudinal index is obtained by computing the mean longitudinal difference between each leg of MDI_(R,i)_ with the corresponding aligned leg of fMDI_(L,i)_. This longitudinal difference between the right leg (resp. left leg) of the reference step from MDI_(R,i)_ and the corresponding aligned leg of the compared step from fMDI_(L,i)_ is denoted $∆R_{\left(i\right)}^{long}$ (resp. $∆L_{\left(i\right)}^{long}$). These longitudinal differences are computed at each height *y* of the leg zone and step interval *i* with Equations (8) and (9).

(8) $$∆R_{\left(i\right)}^{long}\left(y\right)=\underset{x}{mean}(rightleg\left(MDI_{\left(R,i\right)}\left(x,y\right)\right)-rightleg\left(fMDI_{\left(L,i\right)}\left(x+∆R_{\left(i\right)}^{lat}\left(y\right),y\right)\right))$$

(9) $$∆L_{\left(i\right)}^{long}\left(y\right)=\underset{x}{mean}(leftleg\left(MDI_{\left(R,i\right)}\left(x,y\right)\right)-leftleg\left(fMDI_{\left(L,i\right)}\left(x+∆L_{\left(i\right)}^{lat}\left(y\right),y\right)\right))$$

The longitudinal asymmetry index *I_long_*(*i*) is given by the average value of these two distances at each interval *i*:

(10) $$I_{long}\left(i\right)=\underset{y}{mean}(∆R_{\left(i\right)}^{long}\left(y\right)+∆L_{\left(i\right)}^{long}\left(y\right))$$

Thanks to this index for each interval *i*, it is also possible to compute a unique mean value for the whole step:

(11) $$I_{long}=\underset{i=1,\ldots ,10}{mean}I_{long}\left(i\right)$$

### 2.4. Asymmetry Index Based on Skeleton Information 

**Figure 4:**
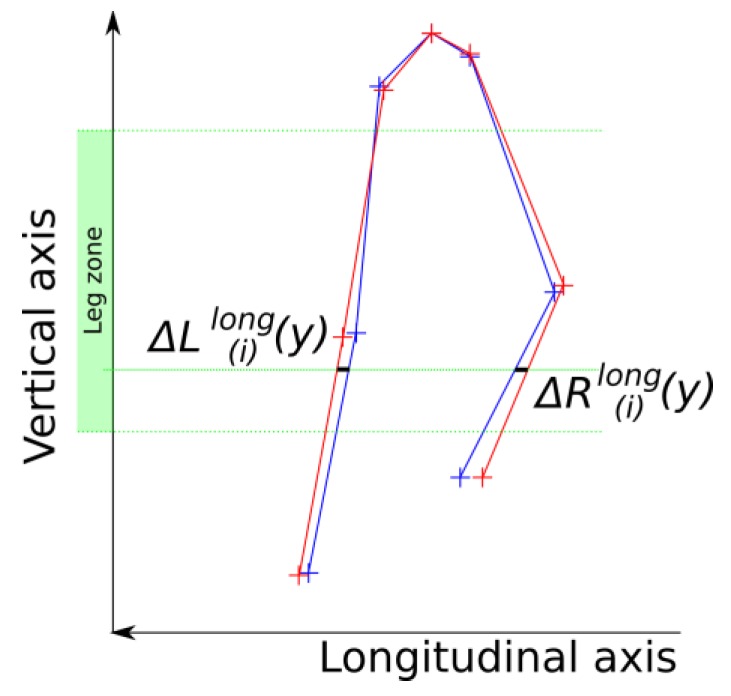
Localization of the longitudinal difference between corresponding legs when using skeleton information.

As previously mentioned, we assumed in this work that skeleton information based on Shotton’s method [14] would not be reliable enough to obtain an accurate asymmetry index. To evaluate this assumption, we applied the method described above with the MSCM to skeleton information provided by the Kinect™ software. We also applied this method to the skeleton information from the reference motion capture system in order to have a ground truth. The computation follows the same scheme as previously presented. Firstly, steps are detected with the distance between knees at a constant estimated knee height, as described in [19]. Each pose is then associated with one of the 10%-long interval *i* of the step. Secondly, the skeleton in each pose is laterally and longitudinally registered thanks to the root joint position, as described above for depth images. Thirdly, poses belonging to the same 10%-long step interval *i* are averaged in order to create a skeleton-based mean gait cycle model. Fourthly, spatial leg positions of poses from corresponding 10%-long intervals are compared to the same method as that previously described with depth images. The pose of the left step is horizontally flipped, and longitudinal differences $∆R_{\left(i\right)}^{long}(y)\;$and $∆L_{\left(i\right)}^{long}(y)\;$are evaluated at each height *y* of the leg zone, as shown in Figure 4.

In this comparison, legs are considered as lines joining two successive joint centers. Linear interpolation between hip, knee and ankle joint locations is used to compute intermediate points along the segment. Finally, the longitudinal asymmetry indices $I_{long}\left(i\right)\;$for each 10%-long interval *i* are computed using Equation (10), and the mean longitudinal asymmetry value for the step $I_{long}$ is computed using Equation (11). The performance of this index is then compared to the one obtained with depth images to evaluate the relevance of our assumption.

### 2.5. Experimentation

We carried out experiments to evaluate the following hypotheses: (1) the longitudinal asymmetry index based on depth images should be reliable enough to quantify asymmetrical gait; and (2) the longitudinal asymmetry index computed thanks to the skeleton information provided by the Kinect™ would lead to significantly less accurate results. Fifteen healthy subjects (12 males, 3 females, 25.3 ± 3.6 years old, 172 ± 7 cm height and 69.4 ± 11.6 kg mass) with no reported clinical asymmetry or gait impairment participated in this study. The institutional ethical review board approved the study.

Each subject walked on a treadmill (LifeFitness F3). The depth images were recorded with a Kinect™ (Microsoft, Redmond, USA) at 30 frames per second with a resolution of 640 × 480 pixels. The Kinect™ was placed 2 m in front of the subject, as shown on Figure 1a. The optical axis of the camera was parallel to the motion direction of the treadmill belt.

To evaluate the accuracy of the longitudinal asymmetry index computed with the skeleton information, we also captured the motion of the subject with an accurate motion capture system (Vicon, Oxford, UK) consisting of 12 infrared cameras capturing motion at 120 Hz. Surface reflective markers were placed over standardized anatomical landmarks, as suggested by the International Society of Biomechanics [20], enabling us to accurately evaluate joint centers, as described in [21]. We used the full body marker set as suggested in [19]. The resulting joint centers could be used to compute the longitudinal asymmetry index as described in Section 2.4, but with reliable and accurate input data.

For each subject, a comfortable speed was selected according to the standard procedure from Holtz *et al.* [22]: the subjects selected their preferred walking speed as the treadmill speed was systematically increased. This comfort speed was then used for all trials. The mean comfortable speed was 1.03 m·s^−1^ ± 0.2.

Each subject performed three trials: one normal gait and two asymmetrical gaits artificially generated by alternatively adding a 5 cm-thick sole under the left and the right foot, as classically suggested in previous works [6,8]. The choice of a 5.0-cm sole was inspired by Bhave *et al.* [23], who indicated that a minimum leg length discrepancy of 4.9 cm was necessary to generate externally visible effects. The duration of a trial after adaptation was set at 6 min with a 2-min adaptation period and a 4-min period of testing to record a large amount of steps. The analysis was automatically carried out on the last 120 gait cycles using the proposed method.

Spatiotemporal gait parameters, such as step length and duration, were measured following the Clark *et al.* [11] method with both Kinect™ skeleton data and motion capture skeleton data on the last 120 gait cycles. Step duration and length were used to compute the reference gait symmetry ratios (*SR_length_* and *SR_duration_*), as suggested by Patterson *et al.* [4]. This reference value from the motion capture system will be used to evaluate the relevance of the index introduced in this paper by assessing the existence of an asymmetry and to evaluate *SR_length_* and *SR_duration_* computed with the Kinect™ skeleton.

### 2.6. Statistical Analysis

A statistical study was conducted to assess the sensitivity of the methods to detect the artificially-introduced asymmetry. The statistical analysis designed by Gouwanda and Senanayake [8] was used because of protocol similarities. One-way ANOVA tests were conducted to examine the significant difference between normal and asymmetric gaits for each index. This one-way ANOVA was applied to each index value for normal, left-deformed and right-deformed gaits for all of the subjects. The alpha level was set at 0.01. Tukey-HSD multiple comparison tests were then carried out to identify the differences between the experiments when the null hypothesis was rejected. This statistical test allows evaluation if the studied index is able to distinguish whether the left (respectively right) deformed gait is different from the normal gait.

This statistical test was conducted from the values of *I_long_(i)* obtained with the three trial conditions (normal, left deformation, right deformation) at each step interval *i*. This is done in order to evaluate the sensitivity of these indices to distinguish the left deformed gait (respectively right) from the normal gait at this step interval.

Moreover, this statistical test was also conducted independently using values of *SR_length_*, *I_long_* and *SR_duration_* obtained with the three trial conditions in order to evaluate the sensibility of these indices to distinguish the left deformed gait (respectively right) from the gait with normal whole step indices.

## 3. Results

The data analysis process worked correctly for all subjects using both our method and the motion capture method. However, a subject has been excluded from the result computed with Kinect™ skeleton data. For one of the trials of this subject, the results were not reliable and an outlier from the others subjects.

### 3.1. SR_duration_ and SR_length_

*SR_length_* and *SR_duration_* computed with Kinect™ skeleton data and the motion capture skeleton are detailed in Table 1. With the Kinect™ skeleton information, no statistical difference exists between these indices for gaits with and without a sole under a foot. However, with the motion capture skeleton data, both *SR_length_* and *SR_duration_* are statistically different (*p* < 0.001) between gait with a sole under one foot and normal gait. These results show that the skeleton information from Kinect™ is not accurate enough to allow *SR_length_* and *SR_duration_* to identify potential adaptations in gait cycles for such a perturbation.

**Table 1:** Results of the symmetry ratios for step duration and for step length (*SR_length_* and *SR_duration_*), computed with the spatio-temporal step information, as suggested by Patterson [4]. The results are reported with the mean value and the standard deviation. Significant statistical results are written in bold and marked with * when *p* < 0.001. *SR_length_* and *SR_duration_* values close to one indicate perfect symmetry of the gait.

Index	Left Deformed Gait	Normal Gait			Right Deformed Gait
Step length symmetry ratio (*SR_length_*) with Kinect™ skeleton					
Left/right SR	0.93 ± 0.11	1.01 ± 0.10			1.10 ± 0.18
Statistical difference	*p* = 0.12		*p* = 0.34		
Step duration symmetry ratio (*SR_duration_*) with Kinect™ skeleton					
Left/right SR	0.99 ± 0.15	1.07 ± 0.18			1.18 ± 0.14
Statistical difference	*p* = 0.17		*p* = 0.49		
Step length symmetry ratio (*SR_length_*) with motion capture skeleton					
Left/right SR	0.87 ± 0.04	1.00 ± 0.04		1.15 ± 0.05	
Statistical difference	*p* < 0.001 *		*p* < 0.001 *		
Step duration symmetry ratio (*SR_duration_*) with motion capture skeleton					
Left/right SR	0.93 ± 0.04	1.00 ± 0.02		1.06 ± 0.04	
Statistical difference	*p* < 0.001 *		*p* < 0.001 *		

### 3.2. Asymmetry Index Based on a Skeleton Estimated with a Kinect™

**Figure 5:**
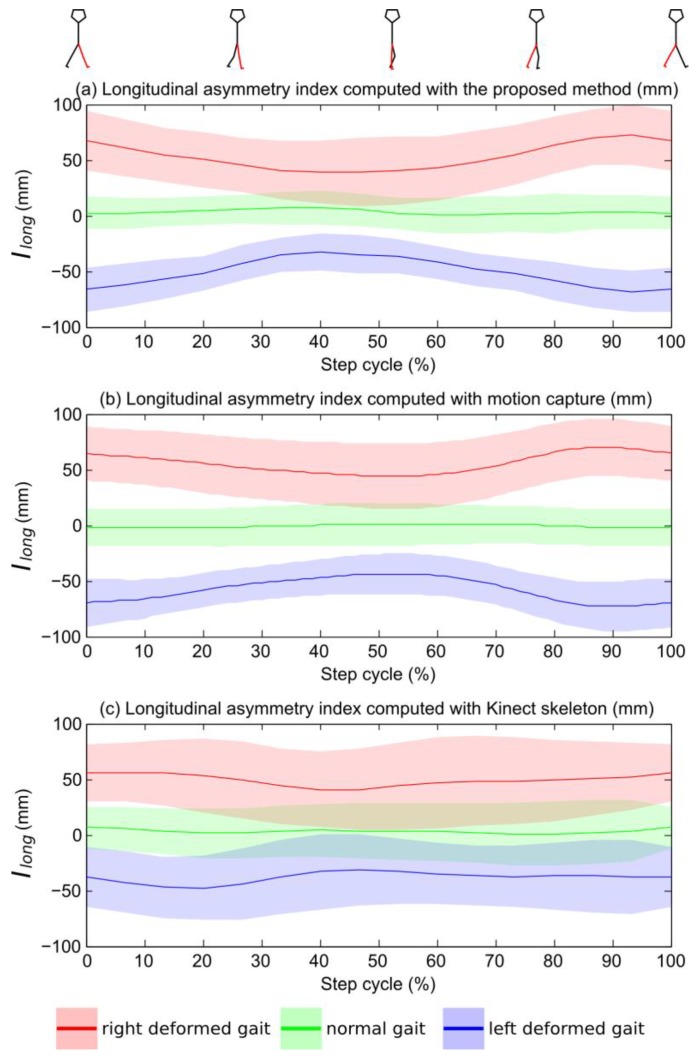
Representation of mean *I_long_(i)* computed for each group (normal, left deformed, right deformed) from raw depth data with our method (**a**) from the skeleton computed with the motion capture data (**b**) and from the skeleton computed with the Kinect™ data. The colored zones define standard deviation intervals. An *I_long_* value close to zero indicates perfect symmetry of the gait.

The results for *I_long_*(*i*) computed at 10 intervals (*i* = 1,…,10) of the step cycle when using skeleton data delivered by the Kinect™ software are presented in Figure 5c. There is no statistical difference between right-deformed gait and normal gait from 50% to 70% of the step. There is no statistical difference between left-deformed gait and normal gait at 50% and 80% and of the step. There is a significant difference for *I_long_* (*p* < 0.001) between the left-deformed gait (resp. right-deformed) and the normal gait, as shown in Table 2. Comparison between the *I_long_*(*i*) computed with the Kinect™ skeleton and the *I_long_*(*i*) computed with accurate motion capture data leads to a correlation coefficient of 0.796. The mean value of *I_long_* was on average 22.5% lower than the reference *I_long_* computed from motion capture for left and right deformation. This means that asymmetry obtained with the Kinect™ skeleton tends to underestimate asymmetry computed with more precise data. Moreover, the standard deviation of the *I_long_* computed with this method was on average 44% higher than reference *I_long_* from motion capture data for both the normal and deformed gait.

**Table 2:** Total asymmetry index results for the average longitudinal asymmetry index (*I_long_*) computed with different methods for normal, left deformed and right deformed gait. The results are reported with the mean value and the standard deviation. Significant statistical results are written in bold and marked with * when *p* < 0.001. An *I_long_* value close to zero indicates perfect symmetry of the gait.

Index	Left Deformed Gait		Normal Gait		Right Deformed Gait			
Average longitudinal asymmetry index (*I_long_*) with the proposed method								
Asymmetry index	−50 ± 20 mm		3 ± 15 mm		53 ± 29 mm			
Statistical difference	*p* < 0.001 *			*p* < 0.001 *				
Average longitudinal asymmetry index (*I_long_*) with motion capture data								
Asymmetry index	−57 ± 20 mm		8 ± 17 mm				56 ± 28 mm
Statistical difference	*p* < 0.001 *			*p* < 0.001 *				
Average longitudinal asymmetry index (*I_long_*) with Kinect™ skeleton data								
Asymmetry index	−38 ± 30 mm	−3 ± 25 mm				48 ± 38 mm		
Statistical difference	*p* < 0.001 *			*p* < 0.001 *				

### 3.3 Asymmetry Index Computed with the Proposed Method Based on Raw Depth Images

The results for *I_long_*(*i*) computed at 10 intervals (*i* = 1,…,10) of the step cycle based on depth images are presented in Figure 5a. At each interval, there is a significant difference (*p* < 0.001) between the left-deformed gait (resp. right-deformed) and the normal gait. There is a significant difference for *I_long_* (*p* < 0.001) between the left-deformed gait (resp. right-deformed) and the normal gait, as shown in Table 2. The correlation between *I_long_*(*i*) computed from the depth images with the proposed method and the *I_long_*(*i*) computed with accurate motion capture data leads to a correlation coefficient of 0.968. The mean value of *I_long_* was on average 9.0% lower than the reference *I_long_* computed from motion capture for left and right deformation. The standard deviations of the *I_long_* computed with this method were on average the same (less than 2.5% of variation) as reference *I_long_* from the motion capture data for both the normal and deformed gait.

These results tend to validate Hypothesis 1: *I_long_* was able to distinguish asymmetrical gaits from symmetrical ones with a high statistical significance. Furthermore, *I_long_(i)* computed with our method has a better correlation with reference data, a lower underestimation of the asymmetry and a lower standard deviation of the results than *I_long_(i)* computed with the Kinect™ skeleton. Moreover, *I_long_(i)* computed with our method is always statistically different for deformed gait *versus* normal gait, whereas *I_long_(i)* computed with the Kinect™ skeleton is not statistically different at several intervals of the step. *I_long_(i)* computed with depth images is then significantly more correlated and sensitive than the one computed with the Kinect™ skeleton, supporting Hypothesis 2. 

Let us now consider the sensitivity of this asymmetry index to the parameters used in the method.

### 3.4. Sensibility of the Leg Zone Estimation

A sensibility study was carried-out regarding the parameters used for the proposed method. These parameters are mainly the knee height, the lower leg zone limit and the upper leg zone limit estimations. 

To this end, we tested various heights for the knee height estimation, ranging from 0.25 H to 0.29 H instead of 0.27 H, which is the parameter used to estimate gait events, as described in [19]. Whatever the height used, the gait event estimation remained unchanged, which shows the robustness of this parameter selection. 

**Figure 6:**
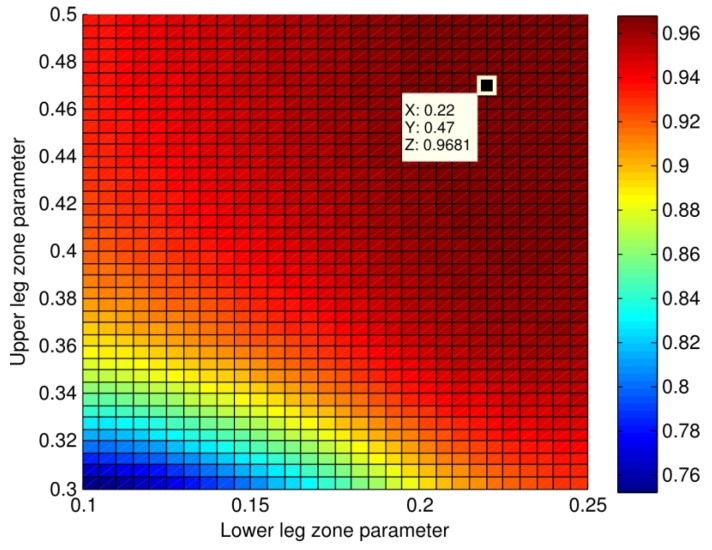
Correlation coefficient between the longitudinal asymmetry index computed from raw depth information with our method and the reference longitudinal asymmetry index computed from motion capture information. The lower leg zone parameter was varied from 0.3 H to 0.5 H, and the lower leg zone was varied from 0.1 H to 0.25 H. The maximum correlation coefficient is 0.968, as shown for the upper leg zone at 0.47 H and the lower leg zone at 0.22 H.

In the same way, we sampled the upper-leg (resp. lower-leg) zone estimation from 0.30 H to 0.50 H (resp. 0.10 H to 0.25 H). The impact on the correlation coefficients between the resulting asymmetry index and the reference value from the motion capture is depicted in Figure 6. Rssesults show that correlation coefficient ranged from 0.75 to 0.968, with a maximum corresponding to the parameters finally selected for the method: 0.22 H for the lower leg zone estimation and 0.47 H for the upper leg zone. The correlation coefficient was higher than 0.9 in 85% of the leg zone parameters tested, which shows the robustness of the method. 

## 4. Discussion

The results show that *I_long_* is a very promising index to detect asymmetry with depth cameras, such as the Kinect™. This index revealed asymmetry globally for the whole step cycle, but also locally for each interval of the step cycle. When estimating spatiotemporal gait parameters with the method proposed in Clark *et al.* [11], we noticed that the feet were sometimes incorrectly located when in contact with the treadmill surface, which partly explains the poor results of the *SR_length_* and *SR_duration_* indices. The indices introduced in this paper are consequently more robust to Kinect™ inaccuracies and errors compared to classical approaches.

The impact of the slope under one foot on *SR_duration_* and *SR_length_* was as described in Gurney [6]. The step length and duration of the shortened leg computed with motion capture data decreased, as shown in Table 1.

We observed that the longitudinal asymmetry index (*I_long_*) changed over time during the step cycle, with a maximum value during the double support phase and a minimum during the swing phase. This is coherent with results reported in the literature by Gouwanda and Senanayake [8]. The *I_long_* variability for normal walking (shaded colored green area in Figure 3a) can be partly explained by the slight natural asymmetry of healthy subjects [2,6]. In the future, we plan to better estimate the sensitivity of this index for subtler asymmetry by using different sole thicknesses under the feet and different modifications.

The method proposed in this paper relied on two main hypotheses, as shown in Section 2.5. Compared to classical symmetry ratios computed using spatio-temporal parameters obtained with the Kinect™ skeleton, our approach was able to highlight significant differences between normal and deformed gaits, as well as classical symmetry ratios computed from spatio-temporal parameters obtained with motion capture. This result supports the first assumption, showing that *I_long_* and *I_long_(i)* computed with depth images are promising gait asymmetry indices based on a simple Kinect™ placed in front of a treadmill. Results for *I_long_(i)* obtained with the Kinect™ skeleton exhibit less satisfactory results, supporting the second assumption. Hence, skeleton estimation inaccuracies lead to less satisfactory asymmetry assessment for the same experimental set-up, supporting the motivation to develop a method based on depth images instead of the Kinect™ skeleton. Furthermore, the correlation coefficient of 0.796 obtained in this study between gait asymmetry measurements using the Kinect™ skeleton and motion capture system is close to the correlation coefficient between Kinect™ and motion capture measurements obtained in [15]. We principally noticed a problem of segmentation of feet. In these cases, parts of the treadmill belt were segmented as belonging to the body. The results could then be increased with a better feet segmentation algorithm. Furthermore, it is possible that the sole under the feet could have disturbed the algorithm. Finally, one of the issues of the Kinect™ skeleton method resides in the need for an open space in front of the subject. Most of the treadmills have a console in front, which cause the Shotton method [14] to fail to reconstruct the skeleton from Kinect™ data. Pfister *et al.* [15] tackle this issue by positioning the Kinect™ on the side of the treadmill with 45 degrees between the optical axis and the progression axis of the treadmill. Furthermore, this could lead to issues for gait asymmetry assessment, as shown by different correlation coefficients regarding the side measured in [15], and as specified in [15], this angle could reduce the algorithm accuracy. However, the backward view on the treadmill is commonly free. To adapt the Shotton method [14], the learning process would have to be carried again in contrast with our method, which could be adapted directly by changing the position of the Kinect™ from the front to the back of the treadmill. We plan to evaluate this new orientation in future work.

The asymmetry index is fully automatic to compute. However, the proposed method relies on two main parameters: (1) the number of intervals that were used to decompose the gait cycle (set to 10 intervals in this paper); and (2) the number of strides used to compute the mean step cycle model (set to 120 cycles in this paper). We evaluated the sensitivity of these parameters by tuning the number of intervals with values ranging from five to 20, and the number of gait cycles from 60 to 180. We did not notice significant changes in the results. We chose 120 gait cycles, which approximately corresponds to a 2-min walking test on the treadmill. Some patients may have difficulties in maintaining a stable walking pattern, and it would be interesting to evaluate to what extended the asymmetry index introduced in this paper could deal with a very small number of steps (down to a single step in the extreme cases). 

## 5. Conclusions

In this paper, we introduced two new gait asymmetry indices based on positional differences between the left and right legs during the gait cycle. They deliver asymmetry information in the two main locomotion planes: lateral and longitudinal. Contrary to many other asymmetry indices, they do not focus on a unique measuring point (compared to force plates or Gaitrite sensors) and can deliver values at different instants during the cycles (compared to SR and other ratios that deliver a unique value for the whole cycle). In this paper, we especially focused on another advantage: robustness when using inexpensive and noisy sensors, such as a Microsoft Kinect™. We have shown that the longitudinal index was able to detect asymmetry caused by a 5 cm sole placed under one shoe, whereas classical indices were not so accurate when using similar data.

This robustness to noisy Kinect™ data is the result of several factors. Firstly, these indices do not rely on a unique point, but on surfaces, which are less sensitive to noise. Secondly, they rely on a mean step cycle model that consists of averaging surfaces of several gait cycles to decrease the influence of noise. In the future, it will be important to evaluate the sensitivity of these indices to the number of cycles used in this model. Especially, one could expect to reach an acceptable accuracy even with one cycle, enabling the use of these indices for over-ground walking.

These indices have been tested on depth images, but it could be possible to adapt them for marker-based systems, such as an optoelectronic motion capture device. Using this standard type of measurement, it would be interesting to compare the performance of this index to previously published ones. Moreover it would be necessary to more carefully analyze the sensitivity of these indices for other types of asymmetry, especially for actual patients with impaired gaits.

A treadmill was used in order to have a relatively constant and ideal distance between the sensor and the subject and to facilitate the acquisition of several gait cycles. However, treadmill walking is substantially different from over-ground walking, although [24] showed that the essential equivalence between treadmill and over-ground walking was enough to allow clinical analysis. Nevertheless, it might be interesting to extend this work to a walking path by using several Kinects along a walkway and by reducing the number of gait cycles used to estimate the MSCM. 

This method focuses on the lower limb movement asymmetry because of its significant contribution to gait movement. It would be very interesting in future work to analyze the spatial difference for the upper leg and for the lower leg separately, as well as to adapt the method for the rest of the body in order to increase the information assessment of gait asymmetry.

Furthermore, it could be interesting to analyze the lateral displacement used to align legs in order to create a lateral asymmetry index. It could be interesting to design a new protocol to induce asymmetry along the lateral axis to evaluate the potential of this index. This index would be interesting because lateral gait asymmetry occurs in some pathologies, such as the circumduction movement in hemiplegic walking.

Being able to obtain relevant asymmetry information even when using a simple Kinect™ sensor is a real advantage in clinical applications: the method is fully automatic, with no marker, no calibration and no manual editing. In addition to screening, it could enable clinicians to perform a follow-up of patients after surgery, treatment (such as joint replacement) or to measure recovery after a stroke. The resulting system is low cost, and easy to use and is a promising tool for a wide range of applications for gait analysis.

## Abbreviation Table

**Abbreviation**	**Type**	**Name**
DI	2D image	Orthographic projection of the 3D surface of the subject
MDI	2D image	Mean depth image
fMDI	2D image	Horizontally-flipped mean depth image
MSCM	Group of MDI	Group of MDI
n	Numeric	Number of step cycles
*i*	Numeric	Interval of the step
${∆}_{z}$	Numeric	Longitudinal global displacement
${∆}_{x}$	Numeric	Lateral global displacement
*c*	Numeric	Step cycle number
*s*	{R,L}	Step side
$∆R_{\left(i\right)}^{lat}$	Vector	Lateral asymmetry at the right leg
$∆L_{\left(i\right)}^{lat}$	Vector	Lateral asymmetry at the left leg
$∆R_{\left(i\right)}^{long}$	Vector	Longitudinal asymmetry at the right leg
$∆L_{\left(i\right)}^{long}$	Vector	Longitudinal asymmetry at the left leg
*Ilong(i)*	Numeric	Longitudinal asymmetry index value at the instant *i* of the step
*Ilong*	Numeric	Mean value of the longitudinal asymmetry index for the step
*SR_length_*	Numeric	Right step length *versus* left step length ratio
*SR_duration_*	Numeric	Right step duration *versus* left step duration ratio
